# Yellow fever cases in French Guiana, evidence of an active circulation in the Guiana Shield, 2017 and 2018

**DOI:** 10.2807/1560-7917.ES.2018.23.36.1800471

**Published:** 2018-09-06

**Authors:** Alice Sanna, Audrey Andrieu, Luisiane Carvalho, Claire Mayence, Philippe Tabard, Marina Hachouf, Claire-Marie Cazaux, Antoine Enfissi, Dominique Rousset, Hatem Kallel

**Affiliations:** 1 Public health direction, Regional Health Agency (Agence régionale de santé Guyane), Cayenne, French Guiana; 2 Regional Unit of Santé publique France (France’s national public health agency), Cayenne, French Guiana; 3 Intensive Care Unit, Cayenne Hospital, French Guiana; 4 APHP-HUPNVS, Department of Anesthesiology and Critical Care, Beaujon Hospital, Clichy, France; 5 National Reference Laboratory for Arboviruses, Institut Pasteur de la Guyane, Cayenne, French Guiana

**Keywords:** yellow fever, surveillance, vaccination, epidemiology, vectorborne disease

## Abstract

French Guiana (FG) is a French overseas region bordering Brazil and Suriname that is considered endemic for yellow fever (YF); vaccination is compulsory for residents and travellers. In August 2017 and 2018, two sporadic YF cases were notified 1 year apart, confirming that sylvatic YF virus circulation is active in the region. YF vaccination coverage should be closely monitored and improved in FG and neighbouring territories and clinicians should be aware of the risk.

In August 2017 and 2018 respectively, sporadic yellow fever (YF) cases were reported in French Guiana (FG). Previously, the last autochthonous case was notified in 1998, in the south-east of FG [1]. Here we describe the clinical and epidemiological features of the two recent YF cases, as well as public health measures implemented.

## Case 1: Enigmatic case from a clandestine gold-mining site, August 2017

The case was a Brazilian woman in her mid-40s who lived and worked in a clandestine, small-scale, gold prospecting site (*garimpo*) in the forested area of the dam lake Petit Saut. In July 2017, she spent ca 1 month in the Amapá state in Brazil, before crossing the border at Oiapoque about 15 days before admission to hospital. She returned to Kourou by sea, continued to travel by land and reached the *garimpo* about 12 days before her admission to Kourou hospital on 7 August 2017 (Figure). She was reported by close contacts at the mining site as initially being either healthy or mildly ill (divergent statements were given). Approximately 5 days before hospitalisation, she reported fever, lumbar and abdominal pain, vomiting and a profound asthenia; relatives witnessed haemorrhagic symptoms (macroscopic haematuria and haematemesis).

**Figure:**
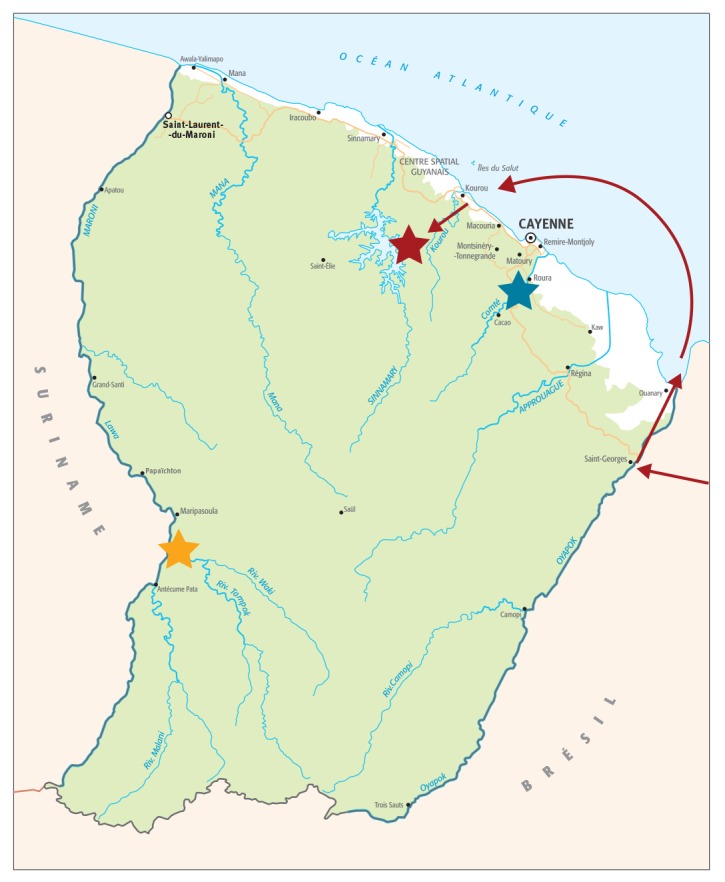
Map with sites of onset of symptoms for notified yellow fever cases and the approximate travel route of the 2017 yellow fever case, French Guiana, 1998‒2018 (n = 3 cases)

Yellow star: 1998 yellow fever case; red star: 2017 yellow fever case; blue star: 2018 yellow fever case.

Modified from IGN 2012 map - Licence ouverte.

The case was admitted at the emergency ward of the Kourou hospital on 7 August, and on 8 August she was transferred to the intensive care unit (ICU) of the hospital in the capital Cayenne due to fulminant hepatitis associated with multi-organ failure. She did not respond to intense supportive therapy and passed away on 9 August. We found no evidence of a former YF vaccination.

A real-time PCR [2] performed on 21 August on a blood sample collected on 8 August allowed confirmation of YF by the arboviruses national reference laboratory (CNR) of the FG Pasteur Institute (IPG) (Table).

**Table:** Laboratory results for infectious aetiologies for yellow fever cases, French Guiana, 2017 and 2018 (n = 2)

Infectious aetiologies	Case 1	Case 2
Yellow fever virus	PCR (blood) pos, IgG neg, IgM pos	PCR (blood) pos, IgG neg, IgM pos
Zika virus	PCR neg, IgG neg, IgM neg	IgG neg, IgM neg
Chikungunya virus	PCR neg, IgG neg, IgM neg	IgG neg, IgM neg
Dengue virus	Antigen NS1 neg, IgG neg, IgM neg	IgG neg, IgM neg
Blood cultures for bacteria	Neg	Neg
Blood cultures for fungi	Neg	NA
Leptospirosis	PCR (urine and blood) neg	PCR (blood) neg
*Plasmodium* spp.	Microscopy neg	Microscopy neg
*Toxoplasma gondii *	IgG pos, IgM neg	NA
*Trypanosoma cruzi*	IgG neg	NA
Herpes simplex virus 1/2	IgG pos, IgM neg	NA
Varicella zoster virus	NA	IgG pos, IgM neg
Cytomegalovirus	IgG pos, IgM neg	IgG pos/neg, IgM neg
Epstein-Barr virus	NA	IgG anti-EBNA pos, IgG anti–VCA pos, IgM anti–VCA neg
Hepatitis A virus	Total Ig pos, IgM neg	Total Ig neg, IgM neg
Hepatitis B virus	HBsAg neg, anti–HBs neg, anti –HBc neg	HBsAg neg, anti–HBs neg, anti–HBc neg
Hepatitis C virus	Total Ig neg	Total Ig neg
Human immunodeficiency virus	Ag P24 neg, serology neg	PCR neg
Human T-lymphotropic virus	Ig neg	NA

EBNA: anti-EBV nuclear antigen-1; Ig: Immuno-globulins; NA: Not available; PCR: polymerase chain reaction; VCA: Viral Capsid Antigen Antibody.

Due to the language barrier and mistrust, likely associated with the clandestine gold-prospecting operation, the epidemiological investigation with the patient’s contacts was complex. Given the uncertainty of the results, in particular travel itinerary and onset of symptoms, and considering different scenarios for the incubation time and sickness duration [3,4], we speculated that the infection might have occurred either in the Amapá state in Brazil or in FG (Petit Saut dam lake area).

### Epidemiological investigation

Passive epidemiological surveillance was strengthened in order to identify possible secondary cases. Moreover, among the locations frequented by the patient while viraemic, only one residential area, in the vicinity of Kourou’s Hospital (range 100 m), was identified where it was possible to perform active case finding. One restaurant and 23 households were identified, of which 15 could be investigated. All of the 41 traced persons were vaccinated against YF, with the exception of two newborns who were asymptomatic.

## Case 2: European traveller in the Amazonian forest, August 2018

The case was a Swiss man, in his late 40s, who supposedly reached FG by land in April 2018 after a long trip in South America. For several weeks he lived in a forest dwelling near the river Comté (Roura municipality). He was reported to have worked on a trail development in the same area the week preceding symptom onset. He reportedly developed mild symptoms (fever, body aches and myalgia) on 4 August. He first sought medical help the following day and was discharged with the diagnosis of acute dengue-like viral infection. He subsequently developed vomiting and deep prostration, associated with persisting high fever and visited the emergency room of the Cayenne hospital on 7 August. He was admitted to the ICU on 8 August due to renal and hepatic failure. On 9 August, he was transferred to a specialised transplant centre close by Paris in mainland France, to receive a hepatic transplant. Despite this he unfortunately died on 30 August. The patient was not vaccinated against YF.

On 10 August, both RT-PCR and serological tests were performed on a blood sample collected on 8 August by the arboviruses CNR of the IPG [2] confirming the diagnosis YF (Table).

In this case, the transmission was clearly autochthonous and sylvatic, occurring in the north of FG, ca 40 km from Cayenne.

### Epidemiological investigation

Active case finding was carried out in the neighbourhood where the case lived. The area is sparsely populated, and only six potentially co-exposed persons were identified, all of them were vaccinated against YF.

In the area there are, however, several touristic structures, and tourists or other people potentially co-exposed e.g. while participating in outdoor sports in the area will be targeted for passive epidemiological surveillance supported by a reporting device.

### Public health measures

For both cases, soon after YF confirmation, vector control measures were strengthened in the locations where the patients transited while viraemic. Information campaigns encouraging vaccination have been carried out in FG, aimed at the general population and clinicians; this communication has been strengthened towards people living and working in areas attended by the cases.

Already since March 2017, we have solicited inhabitants and partner institutions that regularly work in the Amazonian forest (i.e. Amazonian park, French army or organisations dedicated to the preservation of the environment), and they have not reported any evidence of unusual mortality in the non-human primates (NHP) population; nevertheless, in FG, no structured surveillance system of sylvatic epizootics exists.

Illegal gold prospectors were an at-risk, potentially unvaccinated and hard-to-reach population. They live deep in the forest and are potentially co-exposed to the YF virus. Moreover, working in illegal sites and often targeted by police operations they are difficult to reach by health professionals. When the first case occurred, we collaborated with local health and social mediators to invite members of this community who were active near Petit Saut dam lake, to receive vaccination against YF free of charge in nearby health centres. The Kourou health centre of the French Red Cross reportedly received several *garimpeiros* seeking vaccination in the following weeks (exact figures not available). *Garimpeiros were* advised to seek medical care immediately if they developed consistent symptoms: four consulted for fever with an unknown vaccination status, and tested negative for YF PCR.

## Discussion

French Guiana is a French overseas region bordering Brazil and Suriname. It is the only European territory geographically located in the Amazonian region, and considered endemic for yellow fever (YF) [5]. The confirmation of two human YF cases unusually close in time confirms that sylvatic YF circulation is currently occurring in this region.

In 2017, epizootics and sporadic human cases were observed in the northern part of the nearby state of Pará, Brazil [6]; a recent case in neighbouring Suriname was identified in the Brokopondo lake area [7], less than 100 km away from its border with FG. These data suggest ongoing viral circulation in the wider Guiana Shield region. It is not clear, however, whether this is linked with the major epidemics recently observed in the south-eastern region of Brazil [6,8,9].

As the surrounding territories in the Amazonian region, FG is exposed to the risk of both sylvatic (observed) and urban (potential) YF [10]. A sylvatic circulation of YF virus in FG was documented in 1994–95, in a study showing a seroprevalence of 26–35% among NHP in the Petit Saut lake dam forest [11], and was in favour of cyclic and recurrent epizootics [12]. *Aedes aegypti*, an urban vector for YF virus, is largely present in FG [10]. The last documented evidence of human epidemic transmission in an urban context in FG dates back to 1902 [1,13].

Since 1967, YF vaccination has been compulsory for everyone above 1 year of age living in or travelling to FG (airport vaccination status controls are in place) [14]. The latest regional vaccination coverage estimates available for FG range from 80–90% to more than 95% [15]. These figures are in line with the World Health Organization targets of a 60–80% immunisation coverage to avoid major YF epidemics [16], but are not fully satisfactory considering the regional 95% coverage target.

Vaccination coverage should be improved particularly in persons living and working in the forest, who are exposed during the day to sylvatic vectors [17]. It should also be improved in migrants and other vulnerable populations who are more susceptible to being unvaccinated and live in densely populated urban areas (with a risk of urban local transmission), or participate to illegal activities in the forest (with a risk of sporadic cases or clusters in a sylvatic context). A recent random-sampling, whole-population survey will produce vaccination coverage estimates to assist health authorities in targeting at-risk populations.

Despite the relative rarity of the disease, and the complex tropical epidemiological context, clinicians should take YF into account as differential diagnosis when encountering non-vaccinated patients with fever and symptoms compatible with YF, living in or returning from FG. Additional strengthening of laboratory and clinical vigilance in FG may also allow for the detection of sporadic (and even non-severe) cases, which might otherwise remain undiagnosed: severe (ca 12% of YF virus infections) and deadly cases are likely only the tip of the iceberg [18]. Adherence to timely mandatory reporting will enable public health authorities to implement early control measures, and prevent further spread of the disease.
